# Supplementary material for: City-wide school-located influenza vaccination: A retrospective cohort study
Source: Vaccine. Author manuscript; Available in PMC 2022 Feb 26. (PMC8881995; doi:10.1016/j.vaccine.2021.08.099)
Supplement: Benjamin-Chung supp [file NIHMS1765840-supplement-Benjamin-Chung_supp.pdf]

Supplement 1. Additional details about the Shoo the Flu intervention

The intervention aimed to increase influenza vaccination coverage of elementary schoolchildren with the goal of reducing influenza transmission in elementary schoolchildren and community-wide through indirect effects. Children were eligible for vaccination regardless of their insurance status.

In 2014-15 and 2015-16, the intervention implemented a mass media campaign in Oakland, California that included advertisements in subways, bus shelters, billboards, newspapers, and digital media. Though these promotion efforts were restricted to the Oakland area, some residents of other nearby areas may have been exposed to intervention promotion media.

Supplement 2. Additional study design details

<u>Selection of comparison district</u>

We conducted a retrospective cohort study of KPNC health plan members who lived in the catchment areas of the intervention district and a comparison district. To identify the comparison site, we considered school districts in the San Francisco Bay Area with five or more elementary schools located at least five miles from the intervention district. We used a genetic multivariate matching algorithm [28] to pair-match public elementary schools in the intervention district and each candidate comparison district using pre-intervention school-level characteristics: mean enrollment, class size, parental education, academic performance index scores, California standardized test scores, and the school-level percentage of English language learners and students receiving free lunch at school. We selected West Contra Costa Unified School District (WCCUSD) as the comparison site because it had the smallest average generalized Mahalanobis distance between paired schools [28].

<u>Study size</u>

Since our analysis was limited to the KPNC study population, to assess statistical power, we estimated minimum detectable relative risks assuming a two-sided Type I error of 0.05 (Hayes and Bennett, 1999). We calculated the intraclass correlation coefficient for the household-level clustering for each outcome and accounted for these ICCs in the power calculation. There was at least 80% statistical power to detect the following minimum relative risks: MAARI (0.93), positive influenza test (0.93), influenza hospitalization (0.80), and Oseltamivir prescription (0.63).

Hayes RJ, Bennett S. Simple sample size calculation for cluster-randomized trials. Int J Epidemiol **1999**; 28:319–326.

Supplement 3. Deviations from pre-analysis plan

Although Oseltamivir prescriptions (regardless of whether they were filled) was also a pre-specified outcome, we only present results for filled Oseltamivir prescriptions because each influenza season, fewer than 5 patients who received a prescription did not fill it.

We pre-specified laboratory-confirmed respiratory syncytial virus infection as a negative control outcome, but it did not meet the criterion of at least 10 outcome events per variable.

Supplement 4. Additional analysis details

Our primary analysis pooled across individual influenza vaccination status. Since individuals who were vaccinated for influenza at KPNC were unlikely to have participated in the SLIV intervention, to further isolate the effect of the intervention we performed a pre-specified secondary analysis that stratified by whether individuals were vaccinated for influenza at KPNC. Individual-level vaccination data for vaccinations from sources other than KPNC, including the SLIV intervention, was not available. Individuals not vaccinated for influenza at KPNC may have received the influenza vaccine at school or another source or may have been unvaccinated. As such, estimates among the individuals not vaccinated at KPNC capture both direct effects of SLIV and indirect effects among the unvaccinated. Estimates among individuals vaccinated at KPNC capture potential indirect effects of SLIV on non-participants in the SLIV intervention.

Analyses assumed all missing covariate data were missing completely at random. We also used targeted maximum likelihood estimation results (Laan & Rose, 2011) were similar, so we report generalized linear models results.

van der Laan, M. J. & Rose, S. *Targeted Learning: Causal Inference for Observational and Experimental Data*. (Springer, 2011).

Supplement Figure 1. Definition of total effects and indirect effects in this study

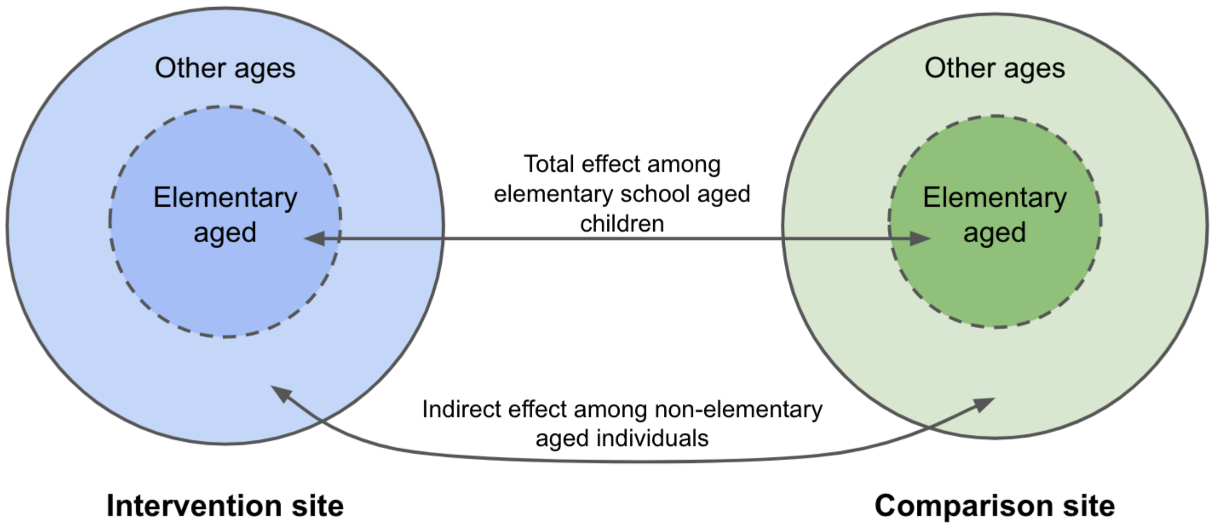

The total effect of the SLIV intervention compares outcomes between elementary school aged children in the intervention vs. comparison site; it combines the direct effect among children who attended intervention schools and the indirect effect among children who did not attend intervention schools. Because it was not possible to link school information to Kaiser patient data, we were not able to decompose total effects into direct and indirect effects among elementary school aged children. The indirect effect among non-elementary aged individuals compares outcomes between individuals in the intervention site (who were not eligible for the SLIV intervention) to those of non-elementary aged individuals in the comparison site.

Supplement Figure 2. Monthly cumulative incidence of each outcome by site

A. Medically attended acute respiratory infection

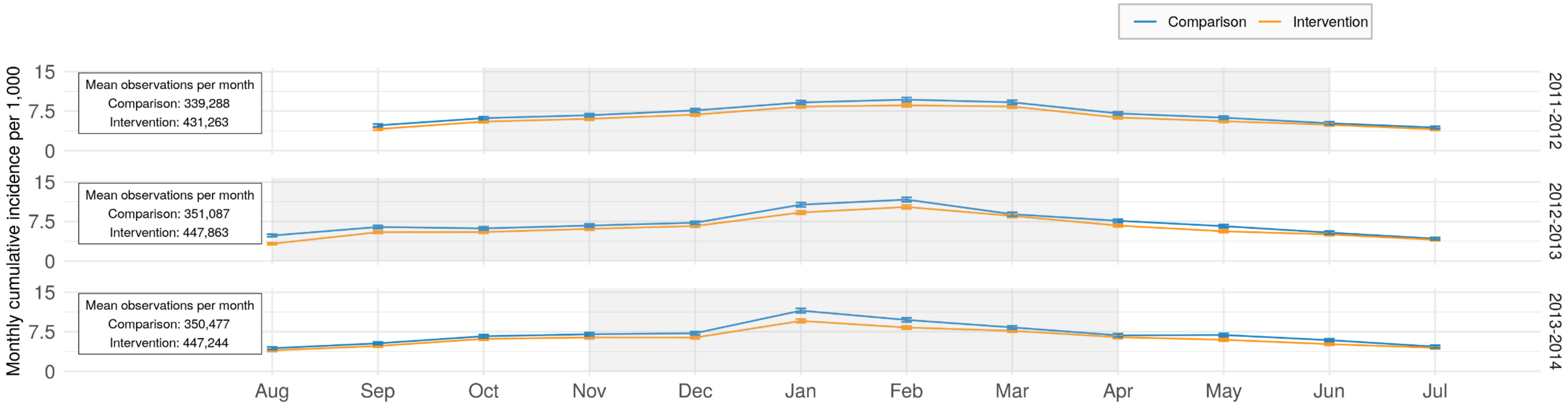

B. Laboratory-confirmed influenza infection among tested individuals

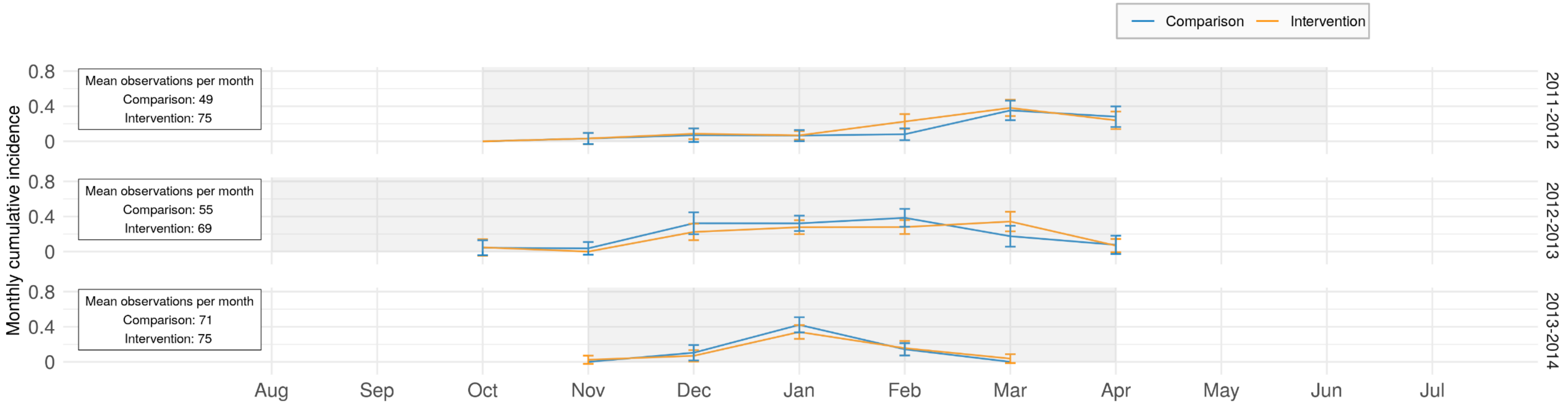

C. Influenza hospitalization

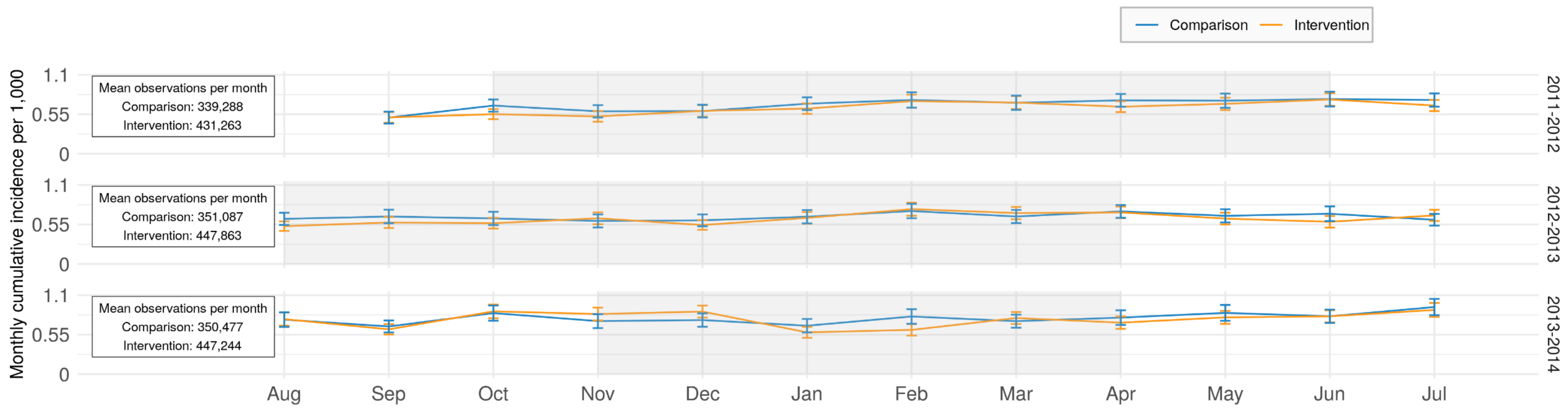

D. Filled Oseltamivir prescriptions

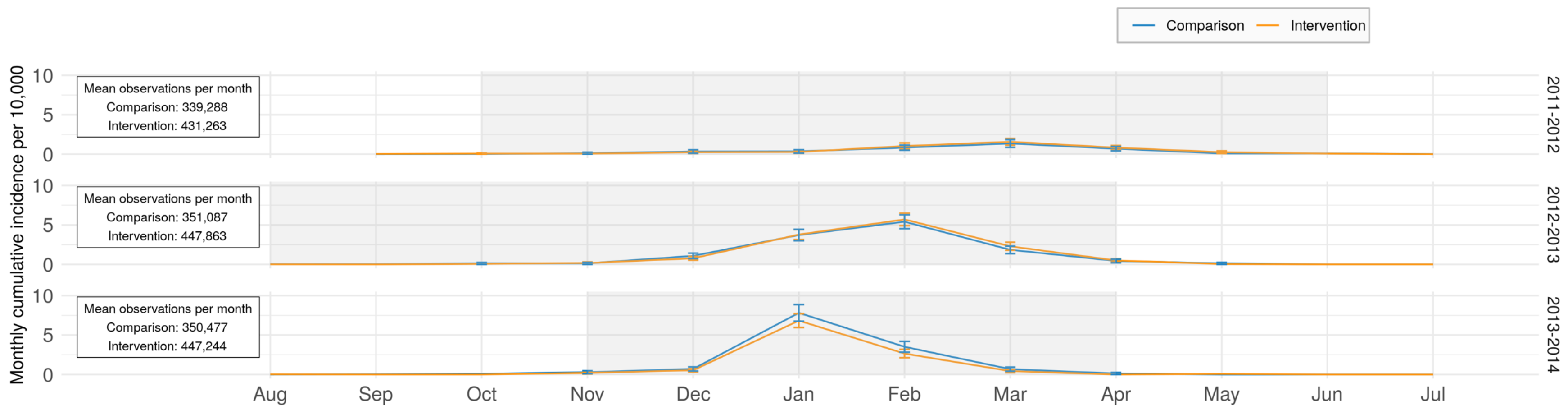

E. Medically attended diarrhea (negative control outcome)

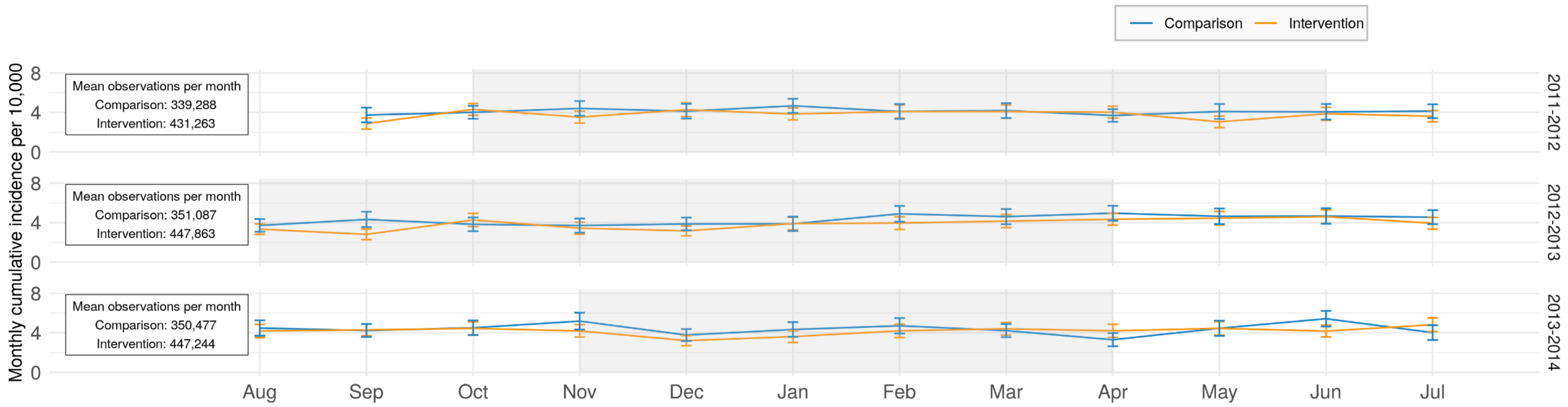

F. Medically attended gastrointestinal illness (negative control outcome)

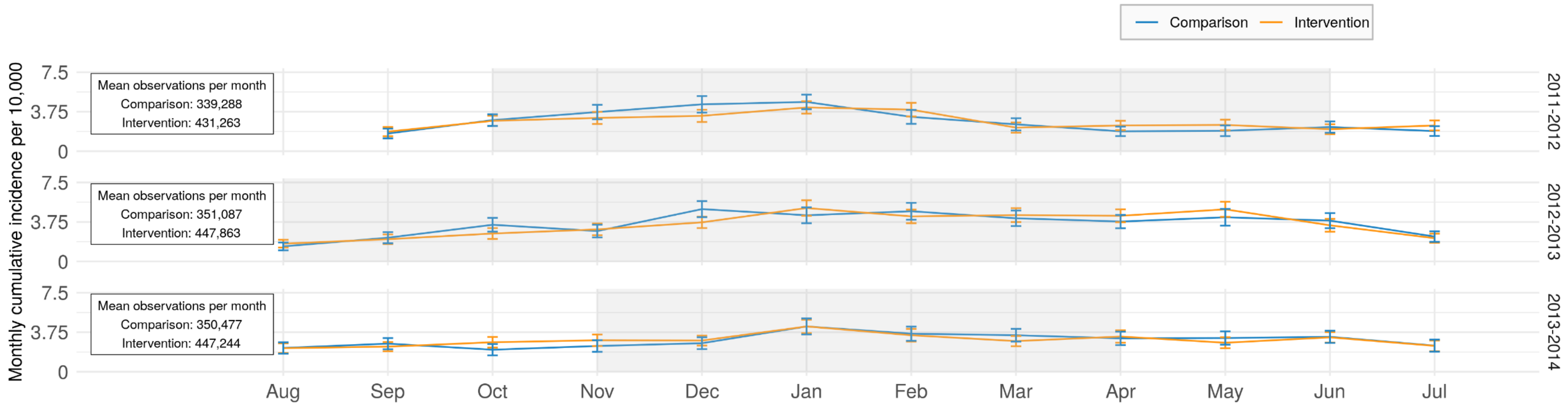

Supplement Figure 3. Influenza vaccination at KPNC by age group and influenza season

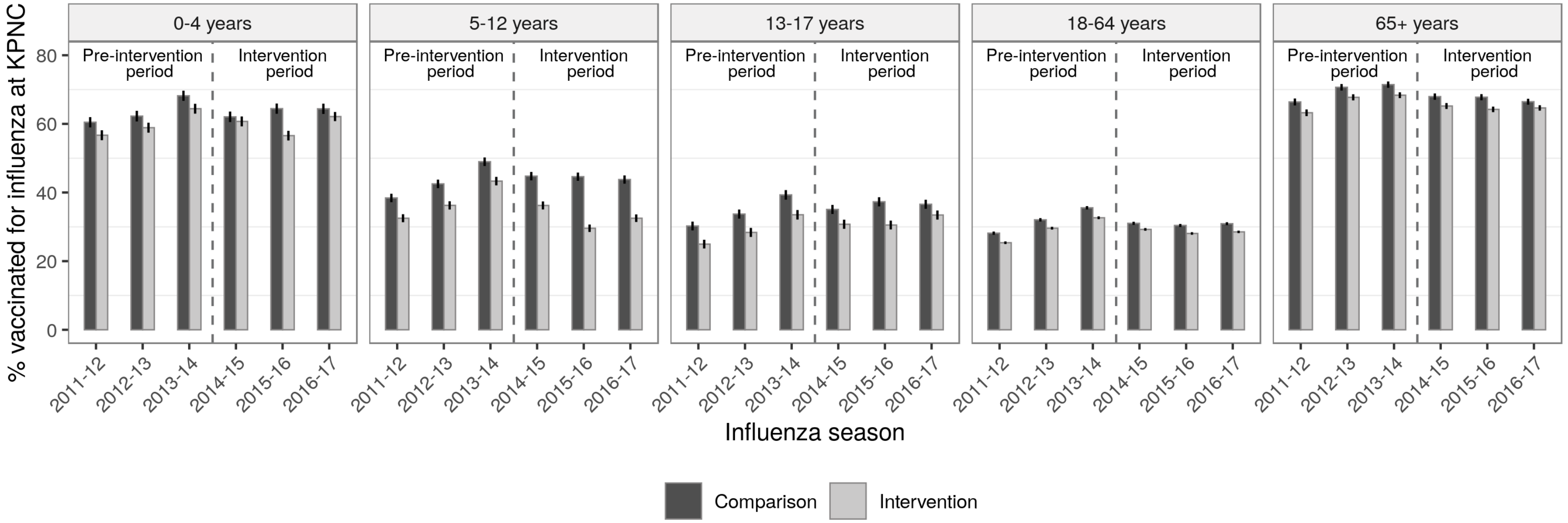

Includes all influenza vaccinations at KPNC between September 1 and August 31 each year.

Supplement Figure 4. Influenza vaccination at KPNC by vaccine type, age group, and influenza season

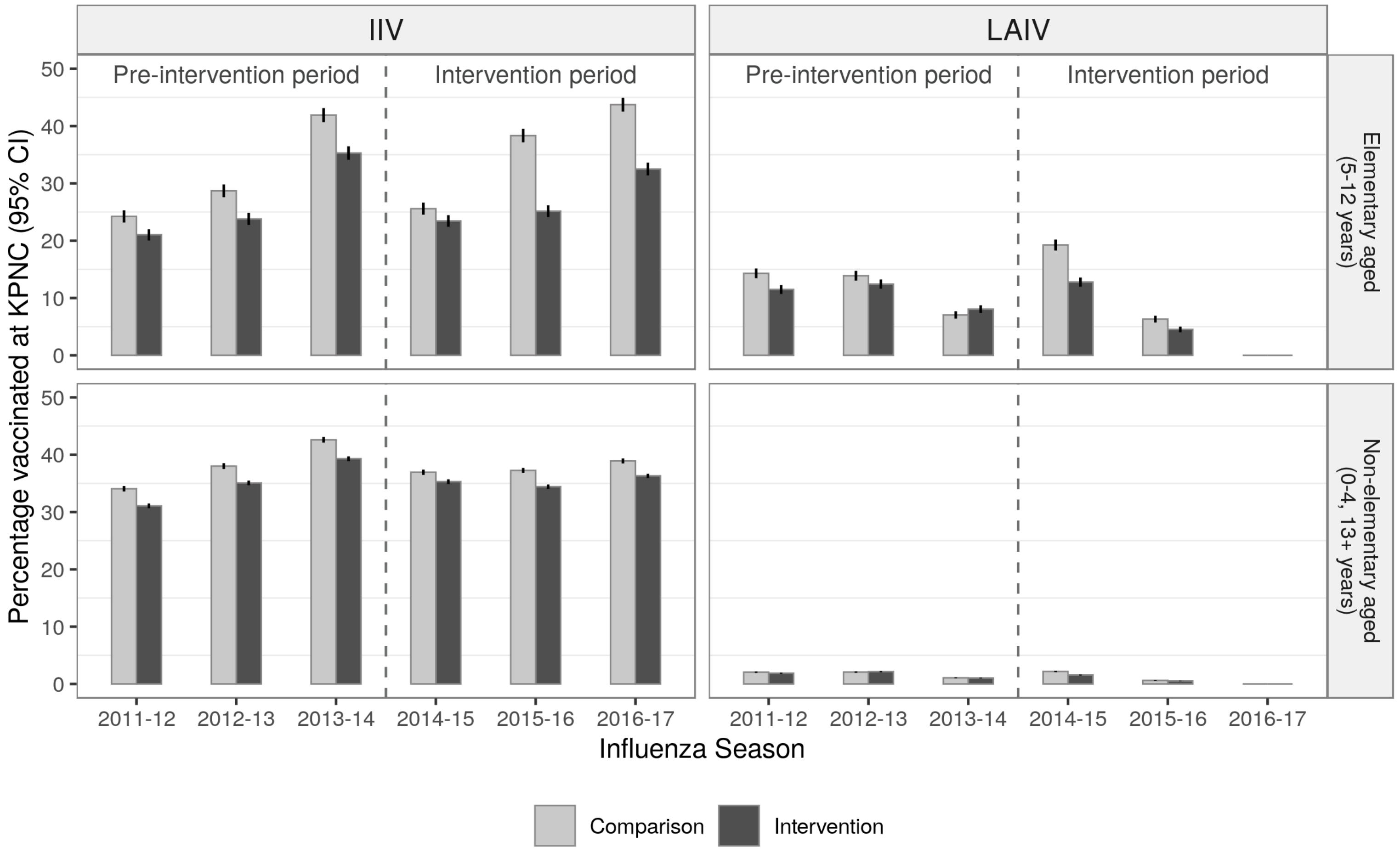

Includes all influenza vaccinations at KPNC between September 1 and August 31 each year.

Supplement Figure 5. Cumulative incidence of each outcome during influenza season by age category and season

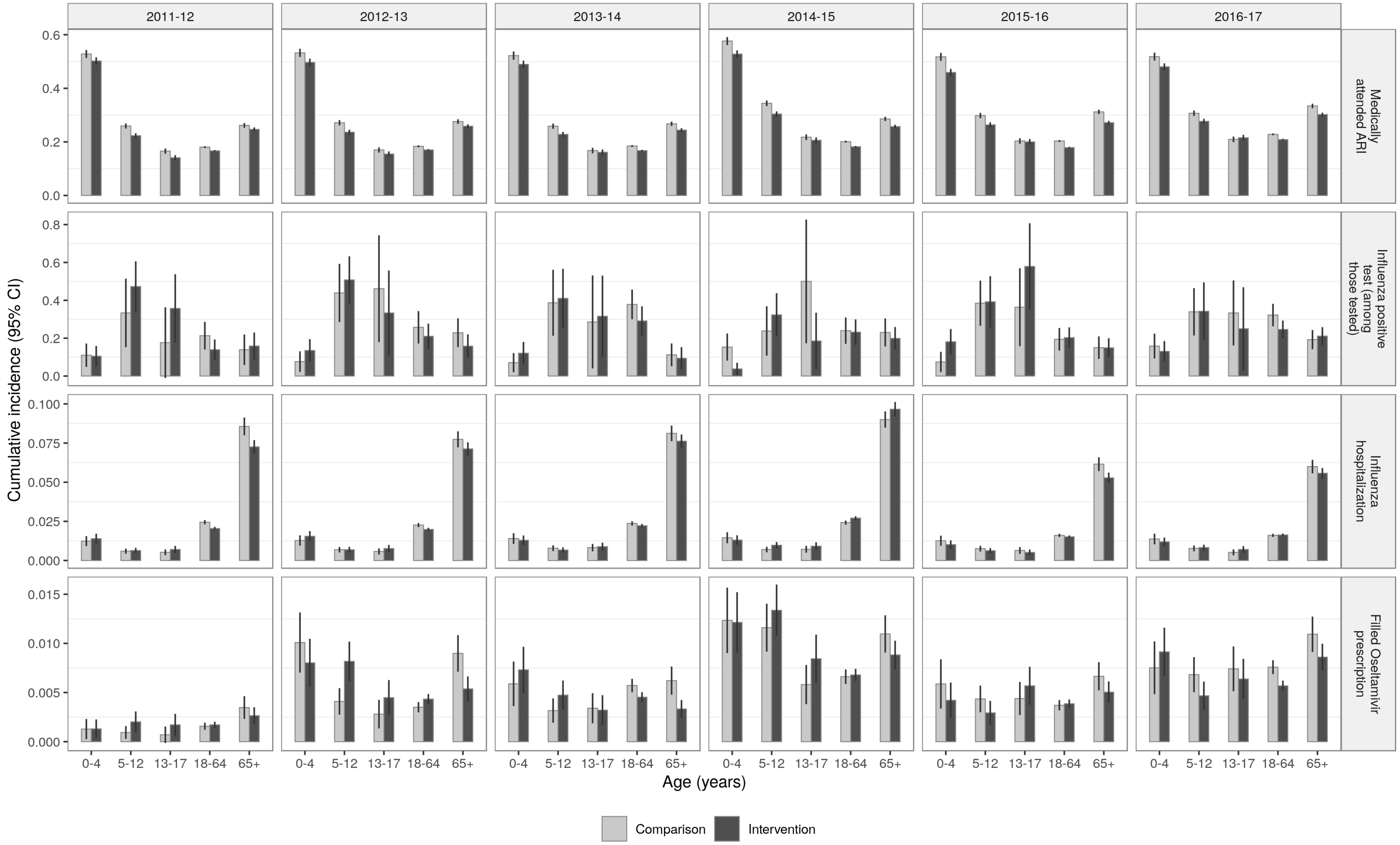

Influenza seasons 2011-12, 2012-13, and 2013-14 were prior to the intervention, and seasons 2014-15, 2015-16, 2016-17 were during the intervention period. Vertical lines in the center of each bar indicate the 95% confidence intervals calculated with robust standard errors to account for clustering at the household level. The standard errors for influenza positive tests did not adjust for clustering because there were very few households with multiple positive individuals.

Cumulative incidence was estimated within influenza season defined based the percentage of medical visits for influenza-like illness in California as reported by the California Department of Public Health. Influenza season started when there were at least 2 consecutive weeks in which the percentage of medical visits for influenza-like illness exceeded 2%, and the season ended when there were at least two consecutive weeks in which the percentage was less than or equal to 2%.

Supplement Figure 6. Difference-in-differences accounting for pre-season differences

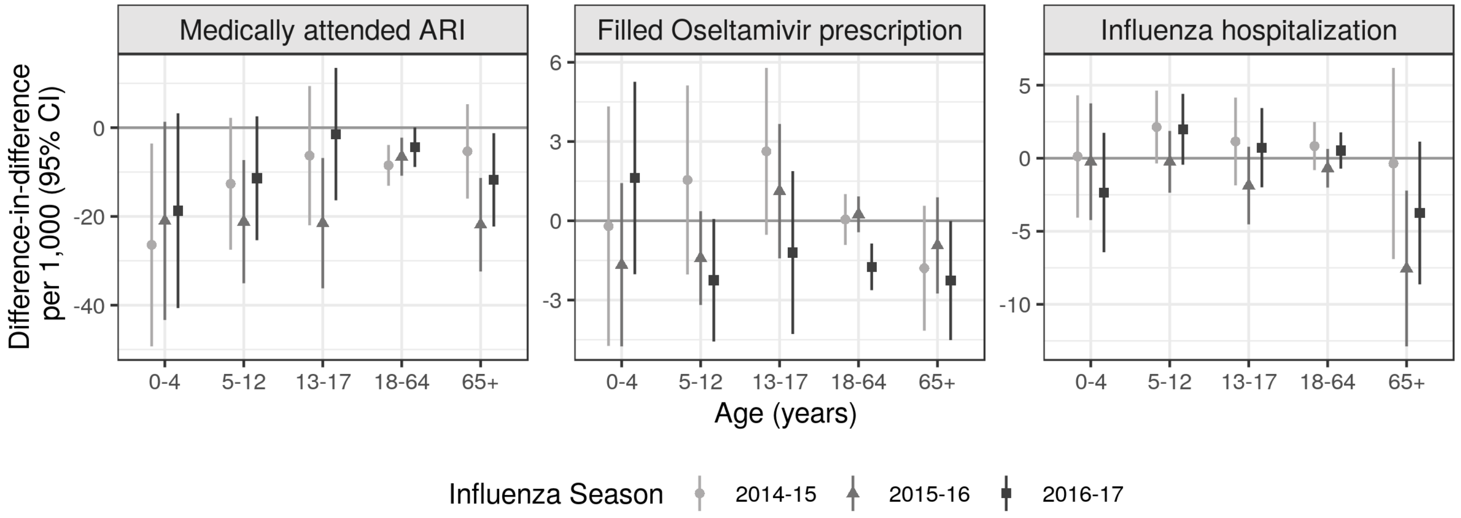

Difference-in-difference comparing the difference in mean outcome in each district in an intervention year compared to the three months prior to each season (May through September). Parameters were estimated using a generalized linear model without covariate adjustment due to data sparsity. Standard errors accounted for clustering at the household level. Estimates in children 5-12 years measure total effects, and estimates in other age groups measure indirect effects. Analyses were restricted to influenza season defined based the percentage of medical visits for influenza-like illness in California as reported by the California Department of Public Health. Influenza season started when there were at least 2 consecutive weeks in which the percentage of medical visits for influenza-like illness exceeded 2%, and the season ended when there were at least two consecutive weeks in which the percentage was less than or equal to 2%.

Supplement Figure 7. Sensitivity analyses with alternative influenza season definitions

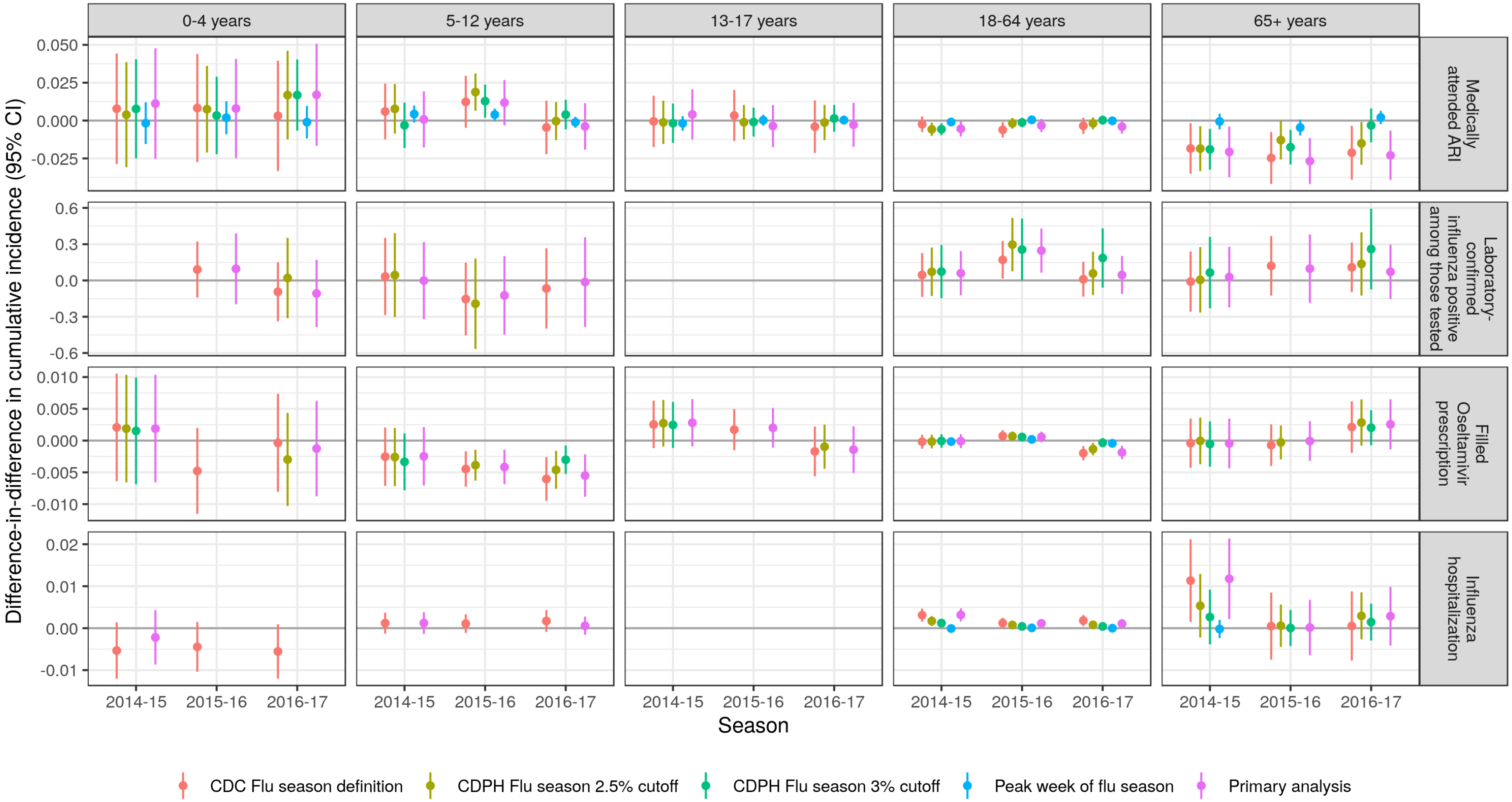

Analyses were restricted to influenza season defined based the percentage of medical visits for influenza-like illness in California as reported by the California Department of Public Health. Influenza season started when there were at least 2 consecutive weeks in which the percentage of medical visits for influenza-like illness exceeded 2%, and the season ended when there were at least two consecutive weeks in which the percentage was less than or equal to 2%. Difference-in-difference estimates compare the difference in mean outcome in each district in an intervention year compared to the three pre-intervention years (2011-2013). Difference-in-difference parameters remove any time-invariant differences between groups (measured or unmeasured). Parameters were estimated using a generalized linear model without covariate adjustment due to data sparsity. Standard errors accounted for clustering at the household level. Estimates are not shown for some outcomes and ages due to sparse data.

Supplement Table 1. International Classification of Diseases, Clinical Modification, Version 9 and 10 (ICD 9/10) codes used to define medically attended acute respiratory illness visits

CODE GROUP	ICD 9 CODE	CONDITION
Viral and chlamydial infection in conditions classified elsewhere and of unspecified site	79.99	Unspecified viral infection
Diseases of ear and mastoid process	382.0*	Acute suppurative otitis media
Diseases of ear and mastoid process	382.4*	Unspecified suppurative otitis media
Diseases of ear and mastoid process	382.9*	Unspecified otitis media
Acute respiratory infections	460.*	Acute nasopharyngitis (common cold)
Acute respiratory infections	461.*	Acute sinusitis
Acute respiratory infections	464.*	Acute laryngitis and tracheitis
Acute respiratory infections	465.*	Acute upper respiratory infections of multiple or unspecified sites
Acute respiratory infections	466.*	Acute bronchitis and bronchiolitis
Pneumonia and influenza	480.*	Viral pneumonia
Pneumonia and influenza	483.*	Pneumonia due to other specified organism
Pneumonia and influenza	484.*	Pneumonia in infectious diseases classified elsewhere
Pneumonia and influenza	485.*	Bronchopneumonia, organism unspecified
Pneumonia and influenza	486.*	Pneumonia, organism unspecified
Pneumonia and influenza	487.*	Influenza
Pneumonia and influenza	488.*	Influenza due to identified avian influenza virus
Chronic obstructive pulmonary disease and allied conditions	490.*	Bronchitis, not specified as acute or chronic
Chronic obstructive pulmonary disease and allied conditions	491.*	Chronic Bronchitis
Chronic obstructive pulmonary disease and allied conditions	493.*	Asthma
General symptoms	780.60	Fever, unspecified
Symptoms involving respiratory system and other chest symptoms	786.2*	Cough
Dyspnea and respiratory abnormalities	786.07	Non-specific wheezing; excludes asthma

Code Group	ICD10 code	Condition
Acute respiratory infections	J00	Acute nasopharyngitis [common cold]
Acute respiratory infections	J01.*	Acute sinusitis
Acute respiratory infections	J02.*	Acute pharyngitis
Acute respiratory infections	J03.*	Acute tonsillitis
Acute respiratory infections	J04.*	Acute laryngitis and tracheitis
Acute respiratory infections	J05.*	Acute obstructive laryngitis [croup] and epiglottitis
Acute respiratory infections	J06.*	Acute upper respiratory infections of multiple and unspecified sites
Acute respiratory infections	J20.*	Acute bronchitis
Acute respiratory infections	J21.*	Acute bronchiolitis
Chronic obstructive pulmonary disease and allied conditions	J40.*	Bronchitis, not specified as acute or chronic

Chronic obstructive pulmonary disease and allied conditions	J41.*	Simple and mucopurulent chronic bronchitis
Chronic obstructive pulmonary disease and allied conditions	J42.*	Unspecified chronic bronchitis
Chronic obstructive pulmonary disease and allied conditions	J44.*	Other chronic obstructive pulmonary disease
Chronic obstructive pulmonary disease and allied conditions	J45.*	Asthma
Diseases of ear and mastoid process	H66.0*	Acute suppurative otitis media
Diseases of ear and mastoid process	H66.4*	Suppurative otitis media, unspecified
Diseases of ear and mastoid process	H66.9	Otitis media, unspecified
Diseases of ear and mastoid process	H67.*	Otitis media in diseases classified elsewhere
Dyspnea and respiratory abnormalities	R06.2	Wheezing
General symptoms	R50.9	Fever, unspecified
General symptoms	R68.83	Chills (without fever)
Pneumonia and influenza	A37.01	Whooping cough due to Bordetella pertussis with pneumonia
Pneumonia and influenza	A37.11	Whooping cough due to Bordetella parapertussis with pneumonia
Pneumonia and influenza	A37.81	Whooping cough due to other Bordetella species with pneumonia
Pneumonia and influenza	A37.91	Whooping cough, unspecified species with pneumonia
Pneumonia and influenza	B25.0	Cytomegaloviral pneumonitis
Pneumonia and influenza	J09.*	Influenza due to certain identified influenza viruses
Pneumonia and influenza	J10.*	Influenza due to other identified influenza virus
Pneumonia and influenza	J11.*	Influenza due to unidentified influenza virus
Pneumonia and influenza	J12.*	Viral pneumonia, not elsewhere classified
Pneumonia and influenza	J13.*	Pneumonia due to Streptococcus pneumoniae
Pneumonia and influenza	J14.*	Pneumonia due to Hemophilus influenzae
Pneumonia and influenza	J15.*	Bacterial pneumonia, not elsewhere classified
Pneumonia and influenza	J16.*	Pneumonia due to other infectious organisms, not elsewhere classified
Pneumonia and influenza	J17.*	Pneumonia in diseases classified elsewhere
Pneumonia and influenza	J18.*	Pneumonia, unspecified organism
Symptoms involving respiratory system and other chest symptoms	R05	Cough

Supplement Table 2. Number of patients and person-weeks by year and season

	Number of patients	Person-weeks	
		Entire follow-up period	Influenza season
2011-12	175,628	9,436,202	6,801,780
2012-13	226,875	9,623,350	5,861,011
2013-14	229,312	9,748,880	3,991,309
2014-15	240,844	10,503,001	5,981,703
2015-16	254,826	11,138,348	3,069,633
2016-17	269,266	11,500,570	3,640,907

The entire follow-up period was from September 1 to August 31 of the following calendar year. Influenza season was defined based the percentage of medical visits for influenza-like illness in California as reported by the California Department of Public Health. Influenza season started when there were at least 2 consecutive weeks in which the percentage of medical visits for influenza-like illness exceeded 2%, and the season ended when there were at least two consecutive weeks in which the percentage was less than or equal to 2%.

Supplement Table 3. Pre-intervention characteristics of study population, general population, and school district population

	Study Population [a]		General Population [b]		School District Population [c]	
	Intervention (%)	Comparison (%)	Intervention (%)	Comparison (%)	Intervention (%)	Comparison (%)
Age (years)						
Under 5	5	6	7	7	--	--
5 to 14	11	14	11	13	--	--
15 to 17	3	4	3	4	--	--
18 to 64	66	62	68	65	--	--
65 +	15	15	11	12	--	--
Race						
Asian / Pacific Islander	15	20	17	19	15	16
Black / African American	28	20	28	19	18	27
Native American	0	0	1	1	0	0
Multiracial	1	1	6	6	1	3
White	35	30	35	37	11	13
Other [d]	--	--	14	18	--	--
Unknown or not reported	20	29	1	1	0	1
Hispanic ethnicity / race [e]	31	43	26	33	54	39
MediCAL enrollee	4	7	2	1	--	--
Primary language spoken is not English	12	14	40	47	43	38

[a] KPNC patient population between September 1, 2013 and August 31, 2014
[b] U.S. 2010 Census data for zip codes overlapping with the study site
[c] California Department of Education data from 2013
[d] This category was not included in the Kaiser or California Department of Education datasets.
[e] KPNC data and the Census consider Hispanic to be an ethnicity, while the California Department of Education data considered it a race. Thus, race category percentages sum to 100% excluding Hispanic for the Study Population and the General Population, and they sum to 100% including Hispanic for the School District Population.

Supplement Table 4. Percentage of elementary school aged patients vaccinated for influenza at KPNC during each year of the study

	Intervention		Comparison		
	**N**	**% (95% CI)**	**N**	**% (95% CI)**	**Difference [a] (95% CI)**
**Pre-intervention period**					
2011-12	8,441	32.5 (31.3, 33.7)	8,562	38.4 (37.2, 39.6)	-5.9 (-7.6, -4.2)
2012-13	8,568	36.3 (35.1, 37.5)	8,552	42.5 (41.3, 43.8)	-6.3 (-8.0, -4.5)
2013-14	8,654	43.3 (42.1, 44.6)	8,537	49.0 (47.7, 50.2)	-5.7 (-7.4, -3.9)
**Intervention period**					
2014-15	8,827	36.2 (35.1, 37.4)	8,710	44.8 (43.6, 46.0)	-8.5 (-10.2, -6.9)
2015-16	9,211	29.6 (28.5, 30.7)	8,968	44.6 (43.4, 45.8)	-15.0 (-16.6, -13.4)
2016-17	9,419	32.5 (31.4, 33.6)	8,942	43.8 (42.6, 45.0)	-11.3 (-12.9, -9.6)

Includes patients aged 5-12 years. Includes all influenza vaccinations at KPNC between September 1 and August 31 each year.

[a] Percentage vaccinated for influenza at KPNC in the intervention site minus percentage in the comparison site. 95% CI accounts for clustering within households.

Supplement Table 5. Unadjusted pre-intervention difference-in-differences

	2014-15		2015-16		2016-17	
	Person-seasons	Estimate (95% CI)	Person-seasons	Estimate (95% CI)	Person-seasons	Estimate (95% CI)
**Medically attended acute respiratory infection per 1,000**						
0-4 years	39,600	-18.1 (-40.8, 4.5)	39,923	-12.5 (-34.2, 9.1)	40,409	-0.9 (-23.1, 21.2)
5-12 years	68,851	-5.3 (-20.1, 9.5)	69,493	3.9 (-8.8, 16.5)	69,675	-0.1 (-12.9, 12.8)
13-17 years	44,327	3.7 (-11.2, 18.7)	44,633	5.9 (-6.7, 18.5)	44,645	6.1 (-7.1, 19.3)
18-64 years	471,767	-8.4 (-12.9, -4.0)	480,303	-2.8 (-6.7, 1.0)	487,592	-4.0 (-8.1, 0.1)
65+ years	104,623	-13.2 (-23.2, -3.2)	106,500	-21.5 (-31.1, -11.9)	108,060	-13.0 (-23.2, -2.9)
**Laboratory-confirmed influenza positive among those tested per 100**						
0-4 years	823	-14.4 (-24.4, -4.5)	765	10.0 (-2.7, 22.7)	810	-5.1 (-16.5, 6.3)
5-12 years	339	-3.0 (-26.2, 20.1)	323	-4.3 (-29.5, 20.8)	303	-6.2 (-34.6, 22.2)
13-17 years	134	--	136	23.5 (-15.3, 62.3)	134	--
18-64 years	1,035	8.3 (-3.7, 20.3)	1,007	13.1 (0.6, 25.7)	1,164	0.7 (-10.3, 11.8)
65+ years	888	-0.3 (-11.9, 11.3)	837	4.1 (-8.3, 16.4)	1,007	5.0 (-5.4, 15.3)
**Filled Oseltamivir prescription per 1,000**						
0-4 years	39,600	-0.2 (-5.0, 4.6)	39,923	-2.5 (-6.0, 0.9)	40,409	1.6 (-2.2, 5.4)
5-12 years	68,851	-0.4 (-4.1, 3.3)	69,493	-3.5 (-5.5, -1.5)	69,675	-4.0 (-6.5, -1.6)
13-17 years	44,327	1.9 (-1.4, 5.3)	44,633	0.6 (-2.1, 3.3)	44,645	-1.4 (-4.5, 1.7)
18-64 years	471,767	0.3 (-0.7, 1.3)	480,303	0.3 (-0.4, 1.0)	487,592	-1.6 (-2.5, -0.7)
65+ years	104,623	0.7 (-1.9, 3.3)	106,500	1.7 (-0.2, 3.6)	108,060	0.8 (-1.6, 3.1)
**Influenza hospitalization per 1,000**						
0-4 years	39,600	-0.9 (-5.4, 3.6)	39,923	-0.4 (-3.8, 3.1)	40,409	-1.1 (-5.0, 2.7)
5-12 years	68,851	1.6 (-0.8, 3.9)	69,493	-0.6 (-2.2, 1.1)	69,675	-0.2 (-2.0, 1.6)
13-17 years	44,327	-0.3 (-3.1, 2.5)	44,633	-1.5 (-3.7, 0.6)	44,645	-1.3 (-3.7, 1.0)
18-64 years	471,767	4.1 (2.5, 5.7)	480,303	1.9 (0.8, 3.0)	487,592	1.7 (0.6, 2.9)
65+ years	104,623	8.0 (2.0, 14.1)	106,500	0.7 (-3.7, 5.2)	108,060	3.0 (-1.7, 7.7)

Person-seasons are the number of individuals in a given season and in all pre-intervention seasons that were included in each difference-in-difference analysis. Estimates in children 5-12 years measure total effects, and estimates in other age groups measure indirect effects. Analyses were restricted to influenza season defined based the percentage of medical visits for influenza-like illness in California as reported by the California Department of Public Health. Influenza season started when there were at least 2 consecutive weeks in which the percentage of medical visits for influenza-like illness exceeded 2%, and the season ended when there were at least two consecutive weeks in which the percentage was less than or equal to 2%. Difference-in-difference estimates compare the difference in mean outcome in each district in an intervention year compared to the three pre-intervention years (2011-2013). Difference-in-difference parameters remove any time-invariant differences between groups (measured or unmeasured). Parameters were estimated using a generalized linear model without covariate adjustment due to data sparsity. Standard errors accounted for clustering at the household level. Estimates are not shown for some outcomes and ages due to sparse data.

Supplement Table 6. Adjusted pre-intervention difference-in-differences

	2014-15		2015-16		2016-17	
	Person-seasons	Estimate (95% CI)	Person-seasons	Estimate (95% CI)	Person-seasons	Estimate (95% CI)
**Medically attended acute respiratory infection per 1,000**						
0-4 years	39,600	-13.5 (-36.1, 9.1)	39,923	-7.4 (-28.9, 14.2)	40,409	4.0 (-18.1, 26.0)
5-12 years	68,851	-1.5 (-16.3, 13.2)	69,493	7.0 (-5.6, 19.6)	69,675	2.9 (-9.9, 15.7)
13-17 years	44,327	4.7 (-10.3, 19.6)	44,633	6.8 (-5.7, 19.3)	44,645	7.0 (-6.2, 20.1)
18-64 years	471,767	-7.7 (-12.1, -3.3)	480,303	-1.6 (-5.4, 2.3)	487,592	-2.0 (-6.1, 2.1)
65+ years	104,623	-12.3 (-22.3, -2.3)	106,500	-20.9 (-30.4, -11.4)	108,060	-12.3 (-22.4, -2.2)
**Laboratory-confirmed influenza positive among those tested per 100**						
0-4 years	823	--	765	--	810	--
5-12 years	339	--	323	--	303	--
13-17 years	134	--	136	--	134	--
18-64 years	1,035	8.9 (-3.0, 20.8)	1,007	--	1,164	0.4 (-10.6, 11.5)
65+ years	888	--	837	--	1,007	5.1 (-5.2, 15.4)
**Filled Oseltamivir prescription per 1,000**						
0-4 years	39,600	-0.1 (-5.0, 4.7)	39,923	--	40,409	--
5-12 years	68,851	-0.3 (-4.0, 3.4)	69,493	--	69,675	-4.0 (-6.4, -1.5)
13-17 years	44,327	2.0 (-1.4, 5.3)	44,633	--	44,645	-1.4 (-4.5, 1.7)
18-64 years	471,767	0.3 (-0.7, 1.3)	480,303	0.3 (-0.4, 1.1)	487,592	-1.5 (-2.4, -0.6)
65+ years	104,623	0.7 (-1.8, 3.3)	106,500	1.7 (-0.2, 3.7)	108,060	0.8 (-1.6, 3.2)
**Influenza hospitalization per 1,000**						
0-4 years	39,600	-0.6 (-5.1, 3.9)	39,923	--	40,409	-0.7 (-4.5, 3.1)
5-12 years	68,851	1.9 (-0.5, 4.2)	69,493	--	69,675	--
13-17 years	44,327	--	44,633	--	44,645	--
18-64 years	471,767	4.3 (2.7, 5.9)	480,303	2.1 (1.1, 3.2)	487,592	2.1 (0.9, 3.3)
65+ years	104,623	7.9 (1.9, 14.0)	106,500	0.6 (-3.9, 5.1)	108,060	2.8 (-1.9, 7.5)

Person-seasons are the number of individuals in a given season and in all pre-intervention seasons that were included in each difference-in-difference analysis. Estimates in children 5-12 years measure total effects, and estimates in other age groups measure indirect effects. Analyses were restricted to influenza season defined based the percentage of medical visits for influenza-like illness in California as reported by the California Department of Public Health. Influenza season started when there were at least 2 consecutive weeks in which the percentage of medical visits for influenza-like illness exceeded 2%, and the season ended when there were at least two consecutive weeks in which the percentage was less than or equal to 2%. Difference-in-difference estimates compare the difference in mean outcome in each district in an intervention year compared to the three pre-intervention years (2011-2013). Difference-in-difference parameters remove any time-invariant differences between groups (measured or unmeasured). Parameters were estimated using a generalized linear model without covariate adjustment due to data sparsity. Standard errors accounted for clustering at the household level. Adjusted DID estimates were not available for some outcomes due to data sparsity.

Supplement Table 7. Unadjusted pre-intervention difference-in-differences among individuals not vaccinated for influenza at KPNC

	2014-15		2015-16		2016-17	
	Person-seasons	Estimate (95% CI)	Person-seasons	Estimate (95% CI)	Person-seasons	Estimate (95% CI)
**Medically attended acute respiratory infection per 1,000**						
0-4 years	15,231	11.2 (-25.3, 47.7)	15,485	8.0 (-24.7, 40.7)	15,355	17.0 (-16.7, 50.7)
5-12 years	41,061	0.8 (-17.8, 19.3)	42,075	11.8 (-3.1, 26.8)	42,010	-3.9 (-19.2, 11.5)
13-17 years	30,115	4.0 (-12.6, 20.6)	30,204	-3.6 (-17.5, 10.3)	30,086	-2.8 (-17.3, 11.7)
18-64 years	328,927	-5.4 (-10.5, -0.3)	336,187	-3.3 (-7.6, 1.1)	340,684	-4.0 (-8.7, 0.6)
65+ years	34,052	-20.7 (-37.4, -4.0)	34,874	-26.8 (-41.9, -11.6)	35,534	-23.0 (-39.2, -6.7)
**Laboratory-confirmed influenza positive among those tested per 100**						
0-4 years	261	--	249	--	253	--
5-12 years	201	--	199	-12.3 (-44.8, 20.1)	189	--
13-17 years	82	--	79	--	80	--
18-64 years	547	6.0 (-12.3, 24.3)	561	24.7 (6.5, 42.9)	650	4.6 (-11.1, 20.2)
65+ years	229	--	209	--	278	7.1 (-15.4, 29.6)
**Filled Oseltamivir prescription per 1,000**						
0-4 years	15,231	1.9 (-6.6, 10.3)	15,485	--	15,355	-1.3 (-8.8, 6.3)
5-12 years	41,061	-2.5 (-7.1, 2.1)	42,075	-4.2 (-6.9, -1.5)	42,010	-5.5 (-8.8, -2.2)
13-17 years	30,115	2.8 (-0.9, 6.5)	30,204	2.0 (-1.1, 5.1)	30,086	-1.4 (-5.1, 2.3)
18-64 years	328,927	-0.1 (-1.2, 1.0)	336,187	0.6 (-0.3, 1.4)	340,684	-1.9 (-2.9, -0.8)
65+ years	34,052	-0.4 (-4.4, 3.5)	34,874	-0.1 (-3.2, 3.1)	35,534	2.6 (-1.4, 6.5)
**Influenza hospitalization per 1,000**						
0-4 years	15,231	-2.2 (-8.7, 4.3)	15,485	--	15,355	--
5-12 years	41,061	1.2 (-1.4, 3.8)	42,075	--	42,010	0.6 (-1.6, 2.7)
13-17 years	30,115	--	30,204	--	30,086	--
18-64 years	328,927	3.2 (1.6, 4.7)	336,187	1.1 (0.1, 2.2)	340,684	1.1 (0.0, 2.2)
65+ years	34,052	11.8 (2.2, 21.4)	34,874	0.2 (-6.5, 6.8)	35,534	2.9 (-4.1, 9.8)

Person-seasons are the number of individuals in a given season and in all pre-intervention seasons that were included in each difference-in-difference analysis. Estimates in children 5-12 years measure total effects, and estimates in other age groups measure indirect effects. Analyses were restricted to influenza season defined based the percentage of medical visits for influenza-like illness in California as reported by the California Department of Public Health. Influenza season started when there were at least 2 consecutive weeks in which the percentage of medical visits for influenza-like illness exceeded 2%, and the season ended when there were at least two consecutive weeks in which the percentage was less than or equal to 2%. Difference-in-difference estimates compare the difference in mean outcome in each district in an intervention year compared to the three pre-intervention years (2011-2013). Difference-in-difference parameters remove any time-invariant differences between groups (measured or unmeasured). Parameters were estimated using a generalized linear model without covariate adjustment due to data sparsity. Standard errors accounted for clustering at the household level. Estimates are not shown for some outcomes and ages due to sparse data.

Supplement Table 8. Unadjusted pre-intervention difference-in-differences among individuals vaccinated for influenza at KPNC

	2014-15		2015-16		2016-17	
	Person-seasons	Estimate (95% CI)	Person-seasons	Estimate (95% CI)	Person-seasons	Estimate (95% CI)
**Medically attended acute respiratory infection per 1,000**						
0-4 years	24,369	-40.8 (-69.9, -11.8)	24,438	-20.8 (-49.1, 7.5)	25,054	-14.6 (-43.0, 13.8)
5-12 years	27,790	-4.7 (-29.4, 19.9)	27,418	2.8 (-19.8, 25.3)	27,665	13.6 (-8.9, 36.0)
13-17 years	14,212	1.3 (-28.9, 31.4)	14,429	22.1 (-3.4, 47.7)	14,559	15.4 (-10.8, 41.7)
18-64 years	142,840	-16.6 (-25.5, -7.6)	144,116	-3.8 (-11.8, 4.1)	146,908	-5.2 (-13.5, 3.1)
65+ years	70,571	-9.4 (-22.1, 3.3)	71,626	-18.7 (-30.9, -6.5)	72,526	-9.2 (-22.0, 3.6)
**Laboratory-confirmed influenza positive among those tested per 100**						
0-4 years	562	--	516	--	557	--
5-12 years	138	--	124	--	114	--
13-17 years	52	--	57	--	54	--
18-64 years	488	6.5 (-8.2, 21.3)	446	--	514	-9.0 (-23.1, 5.1)
65+ years	659	-1.4 (-14.3, 11.5)	628	1.8 (-11.7, 15.2)	729	2.5 (-8.5, 13.4)
**Filled Oseltamivir prescription per 1,000**						
0-4 years	24,369	-1.4 (-7.1, 4.3)	24,438	0.0 (-3.8, 3.9)	25,054	3.4 (-0.7, 7.5)
5-12 years	27,790	3.9 (-2.5, 10.3)	27,418	--	27,665	-1.8 (-5.4, 1.8)
13-17 years	14,212	0.5 (-6.3, 7.3)	14,429	-2.0 (-7.2, 3.1)	14,559	-1.4 (-7.2, 4.4)
18-64 years	142,840	1.4 (-0.8, 3.6)	144,116	-0.4 (-1.8, 1.1)	146,908	-0.9 (-2.7, 0.8)
65+ years	70,571	1.3 (-2.0, 4.6)	71,626	2.6 (0.1, 5.0)	72,526	-0.3 (-3.2, 2.7)
**Influenza hospitalization per 1,000**						
0-4 years	24,369	-0.3 (-6.4, 5.7)	24,438	0.3 (-4.5, 5.0)	25,054	-0.5 (-5.6, 4.7)
5-12 years	27,790	2.5 (-2.0, 7.1)	27,418	--	27,665	--
13-17 years	14,212	-4.4 (-10.9, 2.0)	14,429	--	14,559	--
18-64 years	142,840	6.0 (2.1, 9.8)	144,116	2.7 (0.0, 5.3)	146,908	2.4 (-0.4, 5.3)
65+ years	70,571	6.1 (-1.6, 13.9)	71,626	0.7 (-5.1, 6.4)	72,526	2.5 (-3.5, 8.5)

Person-seasons are the number of individuals in a given season and in all pre-intervention seasons that were included in each difference-in-difference analysis. Analyses were restricted to influenza season defined based the percentage of medical visits for influenza-like illness in California as reported by the California Department of Public Health. Influenza season started when there were at least 2 consecutive weeks in which the percentage of medical visits for influenza-like illness exceeded 2%, and the season ended when there were at least two consecutive weeks in which the percentage was less than or equal to 2%. Difference-in-difference estimates compare the difference in mean outcome in each district in an intervention year compared to the three pre-intervention years (2011-2013). Difference-in-difference parameters remove any time-invariant differences between groups (measured or unmeasured). Parameters were estimated using a generalized linear model without covariate adjustment due to data sparsity. Standard errors accounted for clustering at the household level.

Supplement Table 9. Unadjusted pre-intervention difference-in-differences for negative control outcomes

	2014-15		2015-16		2016-17	
	Person-seasons	Estimate (95% CI)	Person-seasons	Estimate (95% CI)	Person-seasons	Estimate (95% CI)
**Medically attended diarrhea per 1,000 – individuals not vaccinated for influenza at Kaiser**						
0-4 years	15,231	7.5 (-2.3, 17.3)	15,485	7.4 (0.4, 14.3)	15,355	2.2 (-5.7, 10.1)
5-12 years	41,061	-3.8 (-7.3, -0.3)	42,075	-1.0 (-3.9, 1.9)	42,010	2.4 (-0.4, 5.2)
13-17 years	30,115	-0.2 (-3.6, 3.2)	30,204	--	30,086	-1.5 (-5.0, 1.9)
18-64 years	328,927	0.0 (-1.4, 1.5)	336,187	0.3 (-0.9, 1.4)	340,684	-0.1 (-1.2, 1.1)
65+ years	34,052	-2.7 (-8.5, 3.1)	34,874	0.3 (-4.3, 4.9)	35,534	-1.5 (-6.5, 3.4)
**Medically attended gastrointestinal illness per 1,000 – individuals not vaccinated for influenza at Kaiser**						
0-4 years	15,231	14.3 (1.5, 27.2)	15,485	--	15,355	-0.5 (-10.4, 9.3)
5-12 years	41,061	-0.9 (-6.3, 4.6)	42,075	-0.6 (-4.8, 3.7)	42,010	-1.1 (-5.3, 3.1)
13-17 years	30,115	-1.3 (-5.9, 3.3)	30,204	--	30,086	--
18-64 years	328,927	0.4 (-0.9, 1.7)	336,187	0.4 (-0.6, 1.4)	340,684	-0.5 (-1.6, 0.5)
65+ years	34,052	--	34,874	--	35,534	1.5 (-1.6, 4.6)
**Medically attended diarrhea per 1,000 – individuals vaccinated for influenza at Kaiser**						
0-4 years	24,369	-3.4 (-13.3, 6.6)	24,438	1.4 (-6.3, 9.0)	25,054	7.0 (-0.9, 14.8)
5-12 years	27,790	-0.4 (-6.0, 5.1)	27,418	2.8 (-1.2, 6.9)	27,665	1.9 (-2.6, 6.3)
13-17 years	14,212	4.2 (-2.9, 11.3)	14,429	5.0 (-1.0, 11.0)	14,559	--
18-64 years	142,840	-0.7 (-3.3, 2.0)	144,116	-0.2 (-2.4, 1.9)	146,908	-0.3 (-2.6, 1.9)
65+ years	70,571	1.2 (-3.4, 5.9)	71,626	2.1 (-1.6, 5.9)	72,526	1.3 (-2.6, 5.3)
**Medically attended gastrointestinal illness per 1,000 – individuals vaccinated for influenza at Kaiser**						
0-4 years	24,369	-3.1 (-14.7, 8.6)	24,438	-4.3 (-12.7, 4.0)	25,054	-2.2 (-11.4, 7.1)
5-12 years	27,790	2.6 (-5.1, 10.3)	27,418	-5.9 (-11.8, 0.0)	27,665	-7.2 (-13.9, -0.6)
13-17 years	14,212	7.0 (-2.2, 16.1)	14,429	-3.3 (-10.1, 3.5)	14,559	1.4 (-5.5, 8.2)
18-64 years	142,840	2.1 (-0.1, 4.4)	144,116	-0.3 (-2.2, 1.5)	146,908	-0.2 (-2.1, 1.7)
65+ years	70,571	0.6 (-2.3, 3.5)	71,626	0.5 (-1.8, 2.8)	72,526	-2.0 (-4.4, 0.4)

Person-seasons are the number of individuals in a given season and in all pre-intervention seasons that were included in each difference-in-difference analysis. Analyses were restricted to influenza season defined based the percentage of medical visits for influenza-like illness in California as reported by the California Department of Public Health. Influenza season started when there were at least 2 consecutive weeks in which the percentage of medical visits for influenza-like illness exceeded 2%, and the season ended when there were at least two consecutive weeks in which the percentage was less than or equal to 2%. Difference-in-difference estimates compare the difference in mean outcome in each district in an intervention year compared to the three pre-intervention years (2011-2013). Difference-in-difference parameters remove any time-invariant differences between groups (measured or unmeasured). Parameters were estimated using a generalized linear model without covariate adjustment due to data sparsity. Standard errors accounted for clustering at the household level.

Supplement Table 10. Unadjusted pre-intervention difference-in-differences for negative control time period analysis

	2014-15		2015-16		2016-17	
	Person-seasons	Estimate (95% CI)	Person-seasons	Estimate (95% CI)	Person-seasons	Estimate (95% CI)
**Medically attended acute respiratory infection per 1,000**						
0-4 years	15,231	9.1 (-16.9, 35.2)	15,485	-12.8 (-42.2, 16.6)	15,355	7.7 (-22.7, 38.2)
5-12 years	41,061	7.2 (-4.5, 19.0)	42,075	6.7 (-7.3, 20.7)	42,010	-5.2 (-19.8, 9.4)
13-17 years	30,115	6.4 (-4.9, 17.8)	30,204	13.6 (-0.8, 28.0)	30,086	11.8 (-3.1, 26.7)
18-64 years	328,927	2.1 (-1.2, 5.3)	336,187	-8.1 (-12.2, -3.9)	340,684	-1.7 (-6.0, 2.6)
65+ years	34,052	-3.1 (-15.6, 9.5)	34,874	1.5 (-13.7, 16.7)	35,534	0.3 (-14.8, 15.3)
**Influenza hospitalization per 1,000**						
0-4 years	15,231	1.2 (-3.3, 5.7)	15,485	--	15,355	--
5-12 years	41,061	0.5 (-1.6, 2.7)	42,075	--	42,010	-0.9 (-3.2, 1.4)
13-17 years	30,115	--	30,204	--	30,086	--
18-64 years	328,927	1.6 (0.4, 2.8)	336,187	0.1 (-1.2, 1.4)	340,684	0.8 (-0.4, 2.1)
65+ years	34,052	7.8 (-0.2, 15.9)	34,874	-1.1 (-9.1, 7.0)	35,534	1.2 (-6.7, 9.0)

Person-seasons are the number of individuals in a given season and in all pre-intervention seasons that were included in each difference-in-difference analysis. Analyses were restricted to the period outside of influenza season defined based the percentage of medical visits for influenza-like illness in California as reported by the California Department of Public Health. Influenza season started when there were at least 2 consecutive weeks in which the percentage of medical visits for influenza-like illness exceeded 2%, and the season ended when there were at least two consecutive weeks in which the percentage was less than or equal to 2%. Difference-in-difference estimates compare the difference in mean outcome in each district in an intervention year compared to the three pre-intervention years (2011-2013). Difference-in-difference parameters remove any time-invariant differences between groups (measured or unmeasured). Parameters were estimated using a generalized linear model without covariate adjustment due to data sparsity. Standard errors accounted for clustering at the household level.
